# Regulatory T cell therapy in solid organ transplantation: mechanisms, translational progress, and remaining barriers

**DOI:** 10.3389/fimmu.2026.1823841

**Published:** 2026-05-07

**Authors:** Jie Yang, Xusheng Zeng, Ke Jiang, Xiangli Pang, Ai Huang

**Affiliations:** 1 Department of Thoracic Surgery, Union Hospital, Tongji Medical College, Huazhong University of Science and Technology, Wuhan, Hubei, China; 2 Department of Obstetrics and Gynecology, Renmin Hospital of Wuhan University, Wuhan, Hubei, China

**Keywords:** biomarkers, regulatory T cells, solid organ transplantation, translational barriers, transplant tolerance, Treg cell therapy

## Abstract

The long-term outcomes of solid organ transplantation are constrained by alloimmune rejection and the toxicities of lifelong immunosuppression; durable tolerance therefore remains an unmet goal. Regulatory T cells are central mediators of peripheral tolerance, capable of restraining cellular and antibody-mediated rejection while shaping the intragraft inflammatory and repair milieu, supporting a mechanistic rationale for immunosuppression minimization. This review integrates key mechanisms by which Tregs regulate transplant immunity and summarizes evidence for Treg-directed interventions from preclinical studies to early clinical translation across liver, kidney, heart, and lung transplantation. To address the persistent gap between biological promise and inconsistent clinical efficacy, we organize translational barriers using a failure-mode perspective across the therapeutic continuum, encompassing product attributes and manufacturing quality, *in vivo* delivery and persistence, phenotypic stability under inflammatory stress, target engagement, and endpoint sensitivity. In addition, we outline a biomarker-driven evaluation approach centered on quantifiable proof-of-biology and clinically meaningful surrogate endpoints to enable patient stratification, dose/regimen optimization, and risk-controlled immunosuppression minimization. Together, advances in precision engineering, harmonized CMC standards, and mechanism-linked immune monitoring may facilitate reproducible and verifiable tolerance-oriented immunotherapies in solid organ transplantation.

## 1 Introduction

Solid organ transplantation (SOT) is one of the most effective therapeutic strategies for patients with end-stage organ failure, significantly improving both survival rates and quality of life (1, 2). However, despite continuous advances in perioperative management and immunosuppressive regimens, graft immune rejection and long-term complications associated with immunosuppression remain the central challenges limiting long-term transplant success (3). Current immunosuppressive therapies based on calcineurin inhibitors have markedly reduced the incidence of acute rejection (4, 5), yet their ability to improve long-term graft outcomes remains limited. In particular, these regimens fail to effectively prevent chronic immune-mediated injury characterized by progressive structural remodeling and functional deterioration, such as graft vasculopathy and chronic fibrosis. Moreover, long-term use of these agents is associated with severe adverse effects, including infections, malignancies, metabolic disorders, and nephrotoxicity (6). Therefore, achieving “transplant tolerance” (TT)—defined as long-term graft survival and function in the absence of continuous immunosuppression—has become the ultimate goal in the field of organ transplantation.

Regulatory T cells (Tregs) are a critical cellular subset responsible for maintaining peripheral immune tolerance and immune homeostasis, and are characteristically defined by the expression of the forkhead box transcription factor Foxp3, a master regulator that plays an essential role in their development and function (7). Tregs suppress the activation of effector T cells, B cells, and innate immune cells through multiple contact-dependent and contact-independent mechanisms, thereby playing a central role in preventing autoimmunity and limiting excessive immune responses (8). A substantial body of experimental evidence demonstrates that Tregs occupy an indispensable status in the induction and maintenance of graft tolerance, with their abundance and functional status being closely associated with the occurrence and outcomes of transplant rejection (9, 10). Available evidence suggests that, in some recipients of solid organ transplants, long-term graft survival may be associated with increased Treg frequency and enhanced suppressive function, while episodes of acute or chronic rejection have been reported to coincide with reduced Treg numbers or functional deficits (11, 12).

Given the pivotal role of Tregs in the regulation of transplant immunity, Treg-centered cellular therapeutic strategies have gradually progressed from basic research toward clinical translation. In recent years, with advances in cell sorting, ex vivo expansion, and Good Manufacturing Practice (GMP)–grade cell production technologies, multiple therapeutic modalities—including autologous polyclonal Treg infusion (13), donor-specific Tregs (dsTregs) (14), induced regulatory T cells (iTregs) (15), and engineered Tregs (such as chimeric antigen receptor [CAR]-Tregs and T-cell receptor [TCR]-Tregs) (16, 17)—have been successively applied in early-phase clinical studies of liver, kidney, and heart transplantation, demonstrating favorable safety profiles and preliminary efficacy. Beyond exogenous cell infusion, another important approach involves the selective *in vivo* expansion or functional enhancement of endogenous Tregs through pharmacologic agents or cytokines, thereby achieving immunoregulation without direct cell transfer (18). Nevertheless, the application of Treg-based therapies in SOT remains at an exploratory stage. Numerous challenges persist, including issues related to cellular stability, antigen specificity, *in vivo* persistence, optimal timing of administration, and interactions with conventional immunosuppressive agents (19). Moreover, substantial differences among various organ transplants in terms of immunological characteristics, rejection mechanisms, and susceptibility to immune tolerance necessitate that the optimization of Treg therapeutic strategies incorporate organ-specific and individualized considerations.

With the deepening understanding of the mechanisms underlying transplant immune tolerance, Tregs have been increasingly recognized as a critical bridge linking fundamental immunological discoveries with clinical immunomodulatory strategies. In recent years, a variety of Treg-based therapeutic approaches have emerged and have demonstrated favorable safety and potential clinical value in multiple types of SOT. However, their overall application remains at an early exploratory stage, with fragmented evidence and a lack of standardized protocols. This article systematically reviews the mechanisms by which Tregs contribute to immune tolerance in SOT, with a particular focus on summarizing current major Treg-based therapeutic strategies and their clinical research progress in liver, kidney, heart, and lung transplantation. Furthermore, it provides an in-depth analysis of the key bottlenecks and future directions in this field, aiming to offer a reference for promoting the standardized and precision application of Treg cell therapy in SOT.

## 2 Mechanisms of Treg-mediated immune tolerance in solid organ transplantation

### 2.1 Mechanisms by which tregs suppress acute rejection

**Figure 1:**
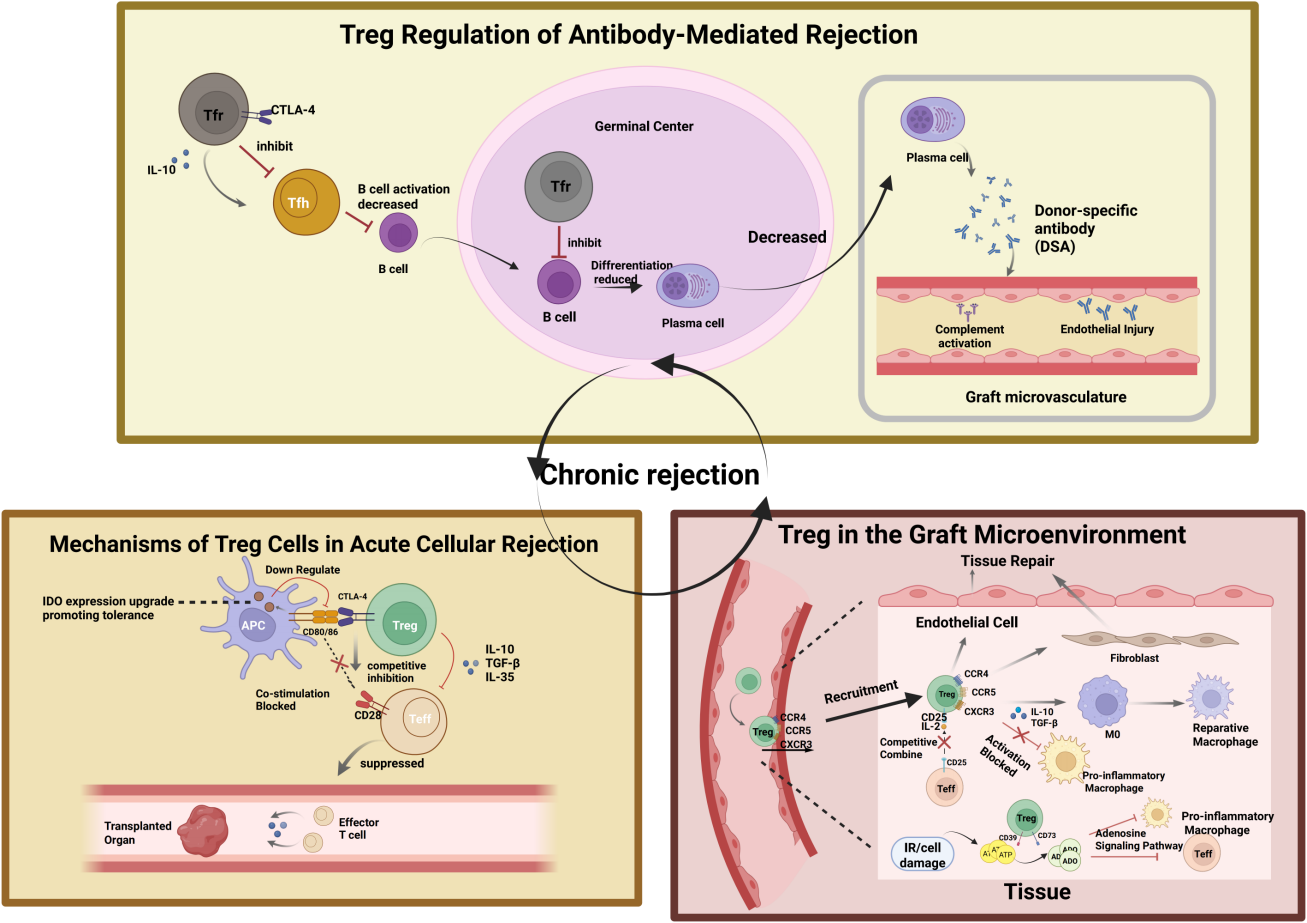
Integrated mechanisms of Treg-mediated immune regulation in allograft rejection. Regulatory T cells control alloimmune responses across multiple stages of transplant rejection. In acute cellular rejection, Tregs suppress effector T-cell activation through CTLA-4–mediated costimulatory blockade, induction of tolerogenic antigen-presenting cells, and secretion of inhibitory cytokines including IL-10, TGF-β, and IL-35. In antibody-mediated rejection, Tregs inhibit T follicular helper cell activity and germinal center B-cell differentiation, thereby reducing plasma cell formation and donor-specific antibody production, ultimately limiting complement activation and endothelial injury. Within the graft microenvironment, Tregs promote immune resolution and tissue repair by modulating macrophage polarization, suppressing local effector responses, and supporting endothelial and stromal recovery. Together, these mechanisms contribute to the prevention of chronic rejection and promote long-term graft survival.

Acute cellular rejection (ACR) is primarily driven by donor-reactive effector T cells, including CD4^+^helper T cells (Th1 and Th17) and cytotoxic CD8^+^T lymphocytes (20). These cells infiltrate the graft and mediate tissue damage through pro-inflammatory cytokines and direct cytotoxic mechanisms. Tregs play an important role in restraining these host-versus-graft and are key contributors to the control of acute rejection. Tregs suppress effector T cell activation through multiple non-redundant mechanisms. The contact-dependent pathway involves the expression of cytotoxic T lymphocyte antigen 4 (CTLA-4), which is highly expressed on Tregs. CTLA-4 competes with CD28 for binding to CD80/CD86 on antigen-presenting cells (APCs), thereby attenuating co-stimulatory signals and inducing a tolerogenic phenotype in APCs (21). Additionally, Tregs secrete immunosuppressive cytokines, including interleukin-10 (IL-10), transforming growth factor-beta (TGF-β), and interleukin-35 (IL-35), which collectively inhibit T cell proliferation, differentiation, and cytokine production (**Figure 1**) (22). Beyond these direct suppressive mechanisms, Tregs may also promote tolerance through linked suppression and infectious tolerance, whereby regulation initiated against one antigen can extend to nearby or related antigens and can foster a broader tolerogenic immune environment. Recent preclinical work has provided further support for these concepts, showing that CAR-Treg cells can mediate linked suppression and induce infectious tolerance in transplantation models (23). In experimental models of SOT, Treg depletion accelerates acute rejection, whereas Treg transfer or *in vivo* expansion extends graft survival and alleviates histological features of rejection. Clinically, increased frequencies of Foxp3^+^Tregs in peripheral blood and graft biopsies of kidney and liver transplant recipients are associated with improved graft function and reduced incidence of acute rejection (24). In contrast, episodes of acute rejection have been reported to coincide with reduced Treg numbers and impaired Treg function, including diminished suppressive capacity, decreased expression of regulatory molecules such as FOXP3, CD25, and CTLA-4, and reduced production of immunoregulatory cytokines such as IL-10 and TGF-β (25).

In addition to directly suppressing effector T cells, Tregs also shape the host-versus-graft response in the early stages by modulating dendritic cell maturation and antigen presentation. Mechanistically, Tregs can reduce the co-stimulatory capacity of antigen-presenting cells through CTLA-4-dependent downregulation of CD80/CD86, including trans-endocytosis of these molecules, thereby limiting dendritic cell-mediated priming of alloreactive T cells. Treg–APC interactions can also induce indoleamine 2,3-dioxygenase expression and promote a tolerogenic dendritic cell phenotype characterized by restrained maturation and reduced T-cell stimulatory activity. In addition, Treg-derived immunoregulatory cytokines such as IL-10 and TGF-β help maintain dendritic cells in a less inflammatory state, further limiting antigen presentation and the initiation of alloimmune responses (**Figure 1**) (26, 27).

### 2.2 Treg regulation of antibody-mediated rejection

Antibody-mediated rejection (ABMR) has emerged as a major cause of late graft loss in SOT and is characterized by the presence of donor-specific antibodies (DSAs), complement activation, and microvascular injury (**Figure 1**). Although ABMR has traditionally been regarded as a B-cell-driven process, accumulating evidence indicates that follicular regulatory T cells (Tfr cells), a specialized subset of regulatory T cells, play an important role in the regulation of humoral alloimmune responses and antibody production (28).

Tfr cells regulate B-cell responses primarily by suppressing follicular helper T (Tfh) cells, which are essential for germinal center formation and the generation of high-affinity antibodies (29). Through mechanisms including CTLA-4 expression and IL-10 secretion, Tfr cells restrain Tfh differentiation and function, thereby limiting B-cell activation, class-switch recombination, and plasma cell generation (30, 31). Tfr cells reside within germinal centers, where they directly suppress B-cell responses and help maintain humoral tolerance (28). Experimental transplantation models have shown that enhancing regulatory pathways involving Tfr cells can reduce DSA levels and attenuate ABMR-associated graft injury (**Figure 1**) (32). Clinically, observational studies in kidney transplant recipients have shown that patients with chronic ABMR exhibit reduced frequencies of Tfr cells in both peripheral blood and graft tissue compared with non-ABMR recipients, and that lower circulating Tfr levels together with a higher cTfh/cTfr ratio are associated with DSA positivity and chronic allograft dysfunction (33, 34). These observations suggest that Treg-based therapies may provide dual benefits in transplantation by suppressing both cellular and humoral immune pathways, although the control of humoral alloimmunity may be particularly related to the activity of Tfr cells.

### 2.3 The role of Tregs in the local graft microenvironment

In addition to systemic immune regulation, Tregs also act within the graft microenvironment, where local immune responses critically influence graft survival. Across solid organ transplantation, intragraft Tregs are thought to limit tissue injury through several shared mechanisms, including chemokine receptor-dependent homing to inflamed tissues, local competition for IL-2, and CD39/CD73-mediated purinergic regulation, although the supporting evidence varies by organ and by rejection context (35–38). At the mechanistic level, Tregs express high levels of CD25 and rely on IL-2-STAT5 signaling to preserve lineage stability and functional fitness, while also competing for local IL-2 and thereby limiting sustained expansion of effector T cells within the graft (36). In parallel, Tregs express ectonucleotidases such as CD39 and CD73, which convert extracellular ATP released during ischemia-reperfusion injury (IRI) and tissue damage into adenosine; this, in turn, suppresses inflammatory responses in effector CD4^+^ and CD8^+^ T cells, as well as in myeloid cells such as dendritic cells and macrophages, thereby promoting a more tolerogenic local milieu (37, 38).

The relevance of these mechanisms differs between acute and chronic rejection. In acute rejection, the available evidence is derived predominantly from experimental animal models, in which intragraft Tregs dampen early inflammatory amplification, restrain effector-cell recruitment, and reduce acute tissue injury within the allograft (39, 40). By contrast, in chronic rejection, Treg-associated effects have been studied mainly in relation to persistent vascular remodeling, macrophage polarization, and progressive fibrosis, with the strongest mechanistic evidence again coming from preclinical models rather than from large human cohorts (12, 36). The organ-specific evidence is also not uniform. In kidney transplantation, most human clinical evidence is observational and suggests that higher intragraft FOXP3/Treg-associated signatures are linked to more favorable long-term graft function and slower progression of chronic allograft injury, although these markers may also reflect ongoing intragraft inflammation and therefore require cautious interpretation (41). In heart transplantation, support for a protective role of Tregs in chronic allograft vasculopathy is currently based mainly on experimental and translational studies, while direct human evidence remains limited (42). In liver transplantation, Tregs are most commonly discussed in the context of the liver’s relatively tolerogenic microenvironment; available human studies associate higher regulatory signatures with operational tolerance, whereas experimental studies support a role for Tregs in maintaining local immune regulation after transplantation (43, 44).

Beyond classical immune suppression, tissue-resident or effector-like Tregs may also contribute to graft adaptation and repair. Through IL-10 and TGF-β-associated interactions with macrophages and structural cells, including endothelial cells, fibroblasts, and parenchymal epithelial cells, Tregs may promote anti-inflammatory or reparative macrophage phenotypes, characterized by reduced pro-inflammatory activity and enhanced regulatory or tissue-repair-associated programs, and thereby influence the balance among inflammation resolution, regeneration, and fibrotic remodeling (35, 45). Collectively, these findings indicate that intragraft Tregs contribute to both the control of acute inflammatory injury and the modulation of chronic vascular and interstitial remodeling, but the strength and nature of the evidence differ substantially across transplanted organs and between human observational studies and animal models.

## 3 Technological approaches for treg-based therapies

### 3.1 *In vitro* expansion and reinfusion of autologous natural Tregs

Autologous natural regulatory T cell (nTreg) expansion and reinfusion represents one of the earliest clinically advanced and most extensively investigated Treg-based therapeutic strategies in SOT (46). This approach involves isolating naturally occurring CD4^+^CD25^+^CD127^low^ Tregs from peripheral blood, expanding them ex vivo under GMP-compliant conditions, and reinfusing them into transplant recipients (47, 48). Polyclonal Tregs exert broad immunosuppressive effects and promote the re-establishment of immune homeostasis by targeting multiple populations of alloreactive effector T cells with diverse antigen specificities. Early clinical trials in kidney (49) and liver transplantation (50) have demonstrated the feasibility and safety of this strategy, with no significant increase in opportunistic infections or malignancies observed. More recent studies have moved beyond demonstrating “safety and feasibility” toward generating quantifiable evidence related to mechanisms of action, dosing, and tissue homing. For example, a prospective randomized clinical trial in kidney transplant recipients with allograft inflammation used biopsy-defined post-transplant inflammatory changes as the primary endpoint and, in parallel, systematically characterized the pharmacokinetics, lineage identity, and tissue trafficking of the infused ex vivo expanded polyclonal Tregs, thereby providing a more mechanistic framework for evaluating Treg therapy beyond conventional safety readouts (51), thereby providing a more verifiable framework for elucidating the underlying mechanisms, optimal timing, and monitoring strategies of this therapy. In parallel, longitudinal follow-up suggests that nTreg reinfusion may support immunosuppression minimization in a subset of recipients, as quantified by successful tapering or withdrawal of antimetabolites, maintenance on reduced or simplified immunosuppressive regimens without biopsy-proven rejection, and stable graft function during follow-up (52–54). Notably, unlike conventional pharmacological agents, Treg-based cellular therapy modulates immune responses without inducing systemic immunosuppression (55).

However, the lack of donor antigen specificity remains a major limitation of polyclonal Treg therapy. Achieving therapeutic efficacy often requires the administration of large cell doses, which raises concerns regarding manufacturing scalability, cost, and *in vivo* persistence (56). Nevertheless, as a pivotal proof of concept, polyclonal Treg therapy has established the clinical safety of Treg-based cellular products and paved the way for the development of more refined antigen-specific and engineered therapeutic approaches.

### 3.2 Donor-specific Tregs

To enhance alloantigen specificity and more effectively suppress host anti-graft immune responses, dsTregs strategies have been progressively developed. Among these, the most clinically advanced approach is donor antigen-reactive Tregs (darTregs). darTregs are typically derived from autologous recipient Tregs and are restimulated and enriched ex vivo using donor APCs or donor-derived peptide antigens, thereby generating a regulatory T-cell population with preferential reactivity toward donor alloantigens (57). Compared with polyclonal Tregs, darTregs exhibit greater suppressive potency at lower cell doses and more effectively inhibit donor-reactive effector T-cell responses (58). Multiple preclinical transplantation models further support the advantages of this strategy, demonstrating that donor-reactive or alloantigen-specific Tregs more effectively prolong graft survival and promote long-term tolerance than polyclonal Tregs (59, 60). The translational significance of these findings lies in the potential to reduce the intensity or duration of conventional immunosuppression, thereby improving long-term graft outcomes (61). Recent early-phase clinical studies have further strengthened signals of feasibility and safety and have incorporated immunosuppression minimization and immune-monitoring readouts into key evaluation frameworks, providing a pathway for darTreg therapy to progress from proof of concept toward reproducible clinical benefit (62). Nevertheless, the broader clinical implementation of darTregs faces practical challenges: their manufacture depends on the availability of donor-derived cells or antigenic materials, resulting in relatively complex production processes and strict time constraints, which are particularly limiting in the setting of deceased-donor transplantation (63). Overall, darTregs represent a critical translational bridge between broad immunomodulation and donor-antigen-directed tolerance, laying the conceptual and technical foundation for subsequent, higher-precision antigen-specific regulatory strategies.

### 3.3 Induced Tregs

iTregs are derived from conventional CD4^+^ T cells under tolerogenic conditions. The classic induction system typically includes TGF-β and IL-2, and can be combined with immune-modulating factors such as retinoic acid or rapamycin to enhance induction efficiency and the suppressive phenotype (64, 65). Compared to the more limited source of nTregs, iTregs offer a more abundant and adaptable cell source for Treg-based therapies. Experimental studies have shown that in transplantation models, iTregs can suppress host-versus-graft disease and extend graft survival (66). However, concerns regarding their lineage stability and phenotypic plasticity have limited their clinical translation. Under pro-inflammatory cytokine conditions, strong TCR stimulation, or environments lacking IL-2 support, iTregs may undergo downregulation of FOXP3 and acquire effector-like functions, thereby posing potential pro-inflammatory risks (67–70). In recent years, significant progress has been made in optimizing strategies to address the critical bottleneck of iTreg “lineage instability in inflammatory environments.” First, because the instability of iTregs is closely linked to incomplete establishment of the Treg-specific epigenetic program—particularly insufficient demethylation of the FOXP3 Treg-specific demethylated region (TSDR/CNS2) and related regulatory elements—considerable effort has been devoted to promoting stable epigenetic remodeling at the FOXP3 locus and across the broader Treg-associated transcriptional network. These approaches aim to reinforce long-term lineage fidelity and suppressive function in iTregs (71–75). Second, to reduce the risk of FOXP3 downregulation and effector differentiation of iTregs in the presence of pro-inflammatory signals and strong immune activation, interventions based on the key regulatory factor RBPJ-NCOR complex identified through functional screening, as well as gene engineering “reinforcement” strategies centered on FOXP3 stabilization, are being used to improve the identity maintenance and functional durability of iTregs in adverse microenvironments (76, 77). Consequently, iTreg therapy has not yet advanced to late-stage clinical trials in SOT. Current research is focused on enhancing iTreg stability through epigenetic regulation (78), metabolic reprogramming (79), and genetic engineering strategies aimed at reinforcing FOXP3 expression and lineage fidelity, including targeted epigenome editing and other non-CAR engineering approaches (75, 80, 81), which may pave the way for safer and more effective clinical applications in the future.

### 3.4 Engineered Tregs

Recent advancements in genetic engineering have driven the development of highly specific Treg products, including CAR-Tregs and TCR-engineered Tregs. These approaches aim to enhance antigen specificity, suppressive potency, and tissue-targeting capabilities. CAR-Tregs can recognize donor-specific HLA molecules or graft-associated antigens independently of major histocompatibility complex (MHC) restriction, enabling precise localization and activation within transplant organs (82, 83). Preclinical studies in heart transplantation models (84) and in skin transplantation models, including A2-CAR Treg studies by Noyan et al. and Boardman et al. (85–87), have shown that CAR-Tregs preferentially accumulate in graft tissues and effectively suppress rejection while preserving systemic immune function. TCR-engineered Tregs, on the other hand, offer an alternative strategy by conferring specificity for donor-derived peptide antigens presented by recipient MHC molecules. Although technically more complex, TCR-engineered Tregs have the potential to provide canonical antigen recognition and sustained regulatory function. Preclinical transplantation studies have shown that TCR-transduced Tregs can enhance targeted immunosuppression by recognizing specific donor antigens and, when combined with adjunctive immunomodulatory regimens, promote long-term survival of heart allografts in murine models (88). These findings support the potential of TCR-Tregs to induce localized immune regulation and transplantation tolerance in solid organ transplantation. However, research on TCR-Tregs remains relatively limited, and further studies are needed to optimize antigen selection, TCR affinity, production feasibility, and long-term stability. Additionally, the safety, durability, and therapeutic efficacy of TCR-Tregs in different solid organ transplant settings must be systematically evaluated prior to clinical translation.

Beyond antigen specificity, engineering strategies also focus on enhancing Treg survival, stability, and migratory capacity through defined molecular modifications. For example, IL-2 receptor engineering has been shown to restore or enhance Treg function under calcineurin inhibitor exposure, thereby improving their functional fitness and persistence. In parallel, chemokine receptor engineering, such as CXCR5 expression, can promote targeted Treg trafficking to secondary and tertiary lymphoid organs, thereby improving site-specific immune regulation (89, 90). Although most engineered Treg approaches in SOT remain at the preclinical stage, they represent a promising direction for the development of precise cell-based therapies.

### 3.5 *In vivo* Treg expansion strategies

Unlike adoptive cell therapy, *in vivo* expansion strategies aim to selectively enhance endogenous Treg populations without ex vivo manipulation. Based on the preferential expression of the high-affinity IL-2 receptor (CD25) on Tregs, low-dose IL-2 therapy has become the most extensively studied approach (91, 92). Current studies indicate that, as an ‘*in vivo* expansion’ strategy, low-dose IL-2 and its engineered derivatives can selectively expand endogenous Tregs *in vivo*. Preliminary signs of efficacy have been observed in exploratory studies of facial transplantation (93) and skin transplantation (94), providing a novel concept for inducing immune tolerance without cell infusion. Meanwhile, evidence from studies in autoimmune diseases and early transplantation trials suggests that low-dose IL-2 generally exhibits a favorable safety profile, effectively expanding and maintaining functional Treg populations while exerting relatively limited effects on the effector T cell compartment. These findings highlight its potential translational value in ‘enhancing immune regulation while avoiding broad immunosuppression’ (95). Currently, modified IL-2 molecules with enhanced Treg selectivity, IL-2/antibody complexes, and IL-2 mutants are under development, with the aim of further improving therapeutic precision (91, 96, 97). Although *in vivo* Treg expansion lacks the antigen specificity characteristic of adoptive Treg therapies, it offers advantages such as operational simplicity, scalability, and compatibility with conventional immunosuppressive regimens. Therefore, IL-2-based strategies may serve as complementary or maintenance therapies to Treg adoptive immunotherapy in transplantation settings.

## 4 Clinical progress of treg therapy in different solid organ transplantations

### 4.1 Liver transplantation

**Figure 2:**
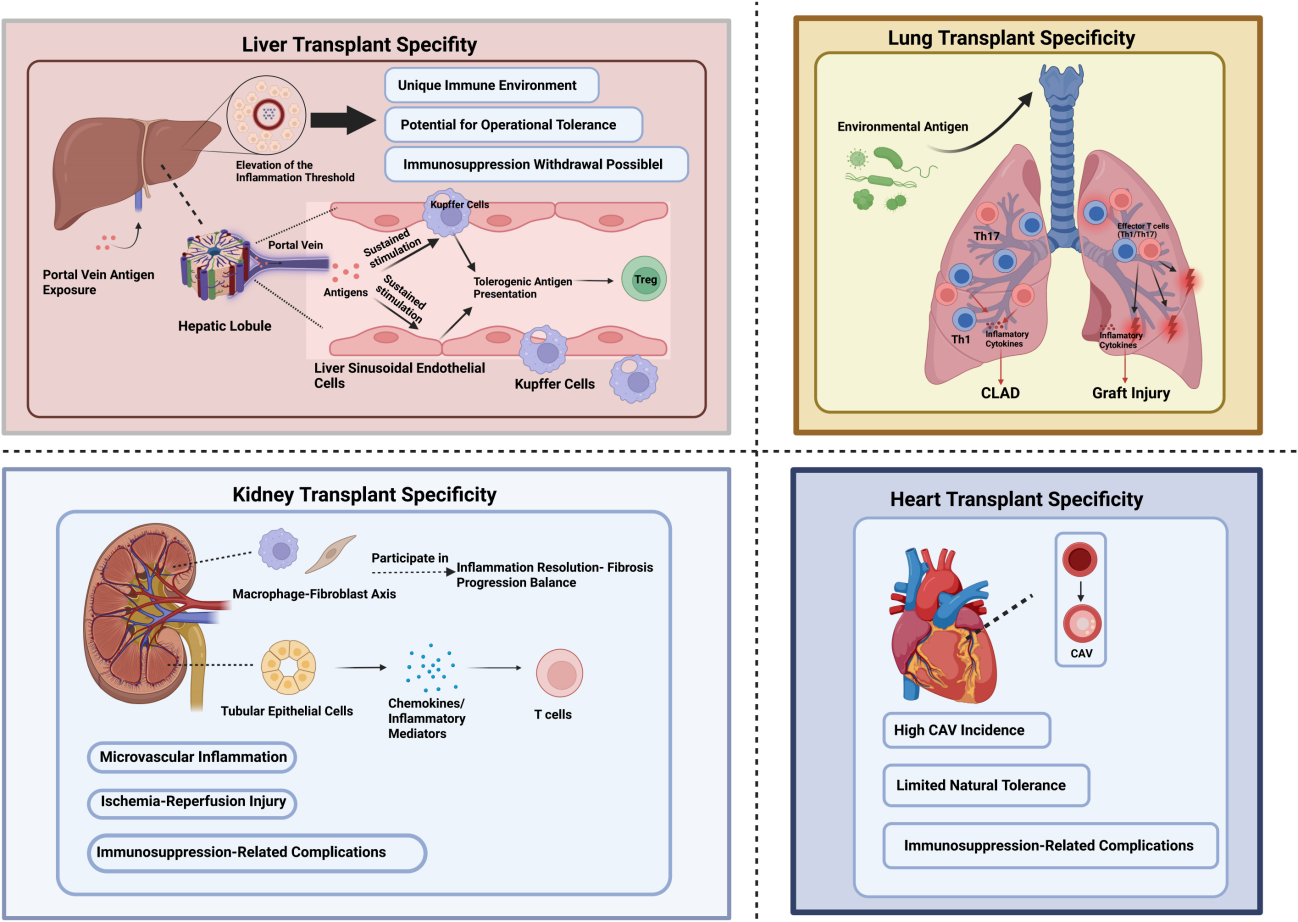
Organ-specific immunological challenges. The liver exhibits intrinsic tolerogenic properties driven by continuous portal antigen exposure and specialized hepatic antigen-presenting cells. Kidney allografts are highly susceptible to inflammatory and fibrotic injury driven by innate and adaptive immune responses. Heart transplantation is associated with a high incidence of cardiac allograft vasculopathy, limited intrinsic immune tolerance, and immunosuppression-related complications. Lung allografts are continuously exposed to environmental antigens, promoting Th1/Th17 responses, inflammatory cytokine production, and effector T cell activation, which contribute to graft injury and chronic lung allograft dysfunction.

In the field of SOT, liver transplantation (LT) represents one of the most advanced clinical applications of Treg-based immunotherapy (98). Historically, the liver has exhibited a unique immunological microenvironment. Owing to continuous exposure to exogenous antigens delivered via portal venous inflow, the liver has developed a tolerogenic antigen processing and presentation network centered on liver sinusoidal endothelial cells and Kupffer cells, which maintains immune surveillance while elevating the threshold for inflammatory activation (99–101). This distinctive feature enables a subset of liver transplant recipients, under strict monitoring, to achieve operational tolerance and immunosuppression minimization or withdrawal (**Figure 2**). Consequently, compared with kidney or heart transplantation, liver transplantation is associated with a relatively higher incidence of functional tolerance following immunosuppressive withdrawal, making it an ideal model for testing tolerance-inducing therapies such as Treg infusion (102). This inherent tolerance provides a biological foundation for translating Treg-based therapies into clinical trials, aiming to reduce lifelong immunosuppression and promote graft acceptance. Tregs have long been recognized as key mediators of immune tolerance, capable of suppressing host-versus-graft reactions, especially within the liver’s distinct immunoregulatory environment (103), where antigen presentation and immune cell interactions differ significantly from other organs (104). Therefore, early clinical and translational evidence suggests that increasing the number of Tregs after liver transplantation may help leverage the liver’s intrinsic tolerogenic tendency to reduce rejection and support immunosuppressive minimization. In particular, a pilot study of donor-specific Treg-based cell therapy in living donor liver transplantation reported successful immunosuppressive withdrawal without clinical rejection in 7 of 10 recipients, providing preliminary support for this concept (105, 106).

A large body of experimental work in liver transplantation models has mechanistically supported the central role of Tregs in regulating transplant outcomes. In a rat tolerant liver transplantation model, tolerant liver allografts exhibited significant upregulation of Treg-associated cytokines, including IL-10 and TGF-β (107), suggesting that Treg-mediated immunoregulatory pathways actively participate in graft acceptance. Further mechanistic studies have shown that exosomes derived from Tregs can inhibit the proliferation of CD8^+^ cytotoxic T lymphocytes and significantly prolong liver allograft survival, thereby providing additional evidence for the functional pathways through which Tregs promote tolerance *in vivo* (**Figure 2**) (108). Building on these findings, several phase I/II clinical studies are currently focusing on the optimization of Treg application in LT.

Beyond these preclinical observations, recent translational studies have focused on improving the feasibility and precision of Treg-based approaches in LT. For example, costimulatory blockade strategies have been shown to reliably expand immunosuppressive Tregs from the peripheral blood of liver transplant recipients without compromising their functional activity (**Table 1**) (109, 110), thereby providing a platform for generating individualized cell products. In parallel, analyses of post-transplant Treg activation and donor responsiveness have further highlighted the complexity of the regulatory networks required for functional tolerance. Emerging studies on HLA-A2-specific CAR-Treg therapy also suggest that engineered Tregs may represent a promising strategy to improve antigen specificity and graft-homing capacity, although these approaches remain at an early exploratory stage and have not yet entered broad clinical application in LT (155).

**Table 1:** Clinical trials of treg-based therapy in solid organ transplantation.

Therapy	ID	Study	Organ	Phase	Study outcome
Adoptive Cell Therapy with Donor Alloantigen-Specific Tregs in Liver Transplantation	UMIN-000015789	Anti-donor regulatory T cell therapy in liver transplantation (106)	Liver	I/IIA	Seven patients successfully discontinued the use of immunosuppressive agents. Three remaining participants developed mild rejection and resumed low-dose immunotherapy
*In Vitro* Expansion and Reinfusion of Autologous Natural Tregs	NCT02166177	Applicability, safety, and biological activity of regulatory T cell therapy in liver transplantation (111)	Liver	I/II	Treg transfer was safe, transiently increased the pool of circulating Tregs and reduced anti-donor T cell responses
Adoptive Cell Therapy with Donor Alloantigen-Specific Tregs in Liver Transplantation	NCT03577431	Ex vivo generation of regulatory T cells from liver transplant recipients using costimulation blockade (110)	Liver	I/II	Costimulatory blockade strategies can stably expand immunosuppressive Tregs from the peripheral blood of liver transplant recipients without compromising their phenotype or functional activity
Expansion of Endogenous Tregs in Kidney Transplantation	NCT02129881	Regulatory T cell therapy is associated with distinct immune regulatory lymphocytic infiltrates in kidney transplants (131)	Kidney	I	Immune cell infiltrates can be observed in transplanted kidneys following adoptive Treg therapy in humans, potentially facilitating T–B cell interactions within the graft that promote local immune regulation
Expansion of Endogenous Tregs in Kidney Transplantation	NCT02088931	Polyclonal Regulatory T Cell Therapy for Control of Inflammation in Kidney Transplants (133)	Kidney	I	Isolation and expansion of Tregs is feasible in kidney transplant patients on immunosuppression. Infusion of these cells was safe and well tolerated
CAR- Treg Therapy	TX200-TR101	Study Design: Human Leukocyte Antigen Class I Molecule A∗02-Chimeric Antigen Receptor Regulatory T Cells in Renal Transplantation (132)	Kidney	I/IIA	TX200-TR101 holds great potential to prevent immune-mediated graft rejection and induce immunologic tolerance after HLA-A∗02-mismatched renal transplantation
*In Vitro* Expansion and Reinfusion of Autologous Natural Tregs	NCT04924491	First-in-human therapy with Treg produced from thymic tissue (thyTreg) in a heart transplant infant (138)	Heart	I/II	Confirm safety of the autologous thyTreg administration and its capacity to restore the Treg pool
Observation	NCT00340951	Regulatory T cells in lung disease and transplantation (150)	Lung	Observational	The the results of this study have not yet been published

Translating these advances into human LT, current clinical studies have primarily focused on safety and feasibility. A landmark pilot study by Todo et al. was the first to suggest that donor-specific Treg infusion may facilitate immune tolerance in human LT: among 10 recipients, 7 successfully discontinued immunosuppressive therapy during follow-up without clinical rejection (**Table 1**) (106). Early open-label phase I studies have likewise reported that Treg infusion is generally safe, with transient increases in circulating Treg levels and a trend toward suppression of anti-donor T-cell responses (**Table 1**) (111). Meanwhile, the ThRIL study (NCT02166177), conducted in London, UK, was designed as a pilot study to evaluate the safety and feasibility of regulatory cell therapy as an adjunct immunomodulatory strategy in liver transplantation, with a second stage intended to assess whether this approach could facilitate immunosuppressive withdrawal while maintaining graft stability. In parallel, a separate low-dose IL-2 study in stable liver transplant recipients on tacrolimus monotherapy showed that IL-2 selectively expanded circulating Tregs, but did not increase the frequency of donor-reactive Tregs, did not promote their trafficking into the liver allograft, and did not facilitate stable tolerance during immunosuppressive withdrawal; rejection events were observed during tapering, This suggests that the strategy of *in vivo* Treg expansion alone has its limitations (112). Together with the pilot study by Todo et al., these findings suggest that Treg-based therapy in LT is clinically feasible and biologically active, but that durable intra-graft persistence, donor specificity, and stable tolerance remain major translational challenges.

In all liver transplantation Treg therapy trials, safety has met acceptable standards. Infusion-related adverse events have been rare and mild, with no significant increase in infectious diseases or malignancies directly attributable to Treg administration (111, 113). Post-infusion immune phenotype analysis has shown a transient increase in the proportion of peripheral Tregs, accompanied by changes in immunoregulatory cytokines. These findings support the continued clinical development of Treg therapy in LT, with a focus on determining the optimal dosage and administration protocols. However, important challenges remain for donor-specific Treg approaches in liver transplantation. Recent human data suggest that donor-reactive Tregs may decline selectively after LT, accompanied by features of generalized Treg activation and senescence, potentially limiting the persistence and efficacy of donor-specific Treg therapy for promoting allograft tolerance (62). In addition, strategies aimed at broadly expanding Tregs, such as low-dose IL-2, have increased circulating Treg numbers but failed to preferentially expand donor-reactive Tregs, promote their accumulation in the liver allograft, or induce operational tolerance (**Table 1**) (112). These findings indicate that, in LT, the numerical expansion of peripheral Tregs alone may be insufficient, and that improving donor specificity, tissue trafficking, persistence, and functional stability remains a major translational challenge. The key challenge of Treg therapy in LT also lies in the lack of validated biomarkers that can reliably predict clinical tolerance and the pathways of graft immune acceptance. Current methods include multiparameter flow cytometry monitoring of Treg phenotype and function (**Table 2**) (114), assessment of donor-reactive immune responses (**Table 2**) (115), and the use of multi-omics approaches to identify signatures associated with tolerance or rejection (**Table 2**) (116). Integrating such biomarker strategies into clinical trials will be crucial for optimizing patient selection and establishing indicators of long-term graft acceptance.

**Table 2:** Summary of key biomarkers and clinical readouts for treg therapy in solid organ transplantation.

Target Organ	Category	Specific Biomarkers/Readouts	Clinical and immunological significance
Liver (LT)	Cellular & Molecular Monitoring	Peripheral Treg frequency; Donor-specific host-versus-graft responses; Multi-omics signatures	Evaluates *in vivo* biological activity, target engagement, and functional operational tolerance
Kidney (KT)	Intragraft Signals	Local infiltrates enriched with IKZF2, IL-10, PD-L1, and TIGIT	Provides direct human evidence of a tissue-specific regulatory microenvironment
	Soluble & Cellular Markers	Th17 response suppression; Cytotoxic CD8+ T cell control	Reflects the attenuation of pro-inflammatory and cytotoxic alloimmunity
Heart (HT)	Mechanistic Biomarkers	IL-35 and IL-10 upregulation; Decreased IFN-γ and TNF-α expression	Strongly correlates with the accumulation and functional stability of intragraft Tregs
Lung (LuT)	Compartmental Profiling	Paired flow cytometry of bronchoalveolar lavage fluid vs. PBMCs; Immune checkpoint expression	Accurately reflects local pulmonary immunoregulatory capacity versus systemic immune status
	Clinical Readouts	Pulmonary function testing; Imaging; Freedom from Chronic Lung Allograft Dysfunction	Ultimate physiological and clinical endpoints for assessing lung graft survival and tolerance
Pan-Organ (Emerging)	High-Dimensional Tracking	TCR repertoire tracking; Donor-derived cell-free DNA; Donor-specific antibodies	Enables early identification of immune escape, humoral risk assessment, and precise patient stratification

In summary, the liver, with its relative intrinsic tolerogenicity and the urgent clinical need to reduce long-term immunosuppressive complications, has become an important platform for Treg immunotherapy trials. Early human studies have confirmed that Treg infusion is feasible and appears to be safe, and in some settings may facilitate immunosuppression minimization. However, recent studies also highlight that donor-specific Treg therapy in LT faces distinctive biological barriers, particularly the loss or inadequate enrichment of donor-reactive Tregs after transplantation. Ongoing and future clinical studies will therefore need to address not only safety and efficacy, but also the optimization of donor specificity, *in vivo* persistence, intrahepatic trafficking, and biomarker-guided patient stratification.

### 4.2 Kidney transplantation

Kidney transplantation (KT) is the most common solid organ transplant worldwide (117, 118), and chronic allograft nephropathy remains the leading cause of long-term graft loss (119). In contrast to the relatively tolerogenic immune bias of the liver, the immunological microenvironment of renal allografts is more susceptible to initiation and amplification by IRI and microvascular inflammation. Renal tubular epithelial cells can participate in the production of chemokines and inflammatory mediators and thereby modulate tissue T-cell responses, while the axis formed by resident macrophages and fibroblasts determines the balance between inflammatory resolution and the progression of fibrosis (**Figure 2**) (120, 121). Meanwhile, although conventional immunosuppressive therapies improve short-term outcomes, they are accompanied by adverse effects such as nephrotoxicity, increased risk of infection, malignancy, and metabolic complications (122), highlighting an urgent need for the development of tolerance-promoting strategies with minimal systemic toxicity. By suppressing effector T-cell activation and promoting anti-inflammatory or reparative myeloid polarization, Tregs may represent a critical endogenous braking mechanism limiting chronic structural remodeling. Mechanistically, this effect is thought to involve IL-10- and TGF-β-associated signals that restrain inflammatory macrophage activation and favor macrophage programs linked to immune regulation, resolution of inflammation, and tissue repair (123). A large body of animal experimental studies has mechanistically confirmed that Tregs play a central role in immune regulation and tolerance induction in KT. In both mouse and rat KT models, Treg deficiency significantly accelerates both acute and chronic rejection, while *in vivo* expansion or adoptive transfer of Tregs effectively prolongs graft survival. Mechanistic studies have shown that Tregs can inhibit effector T cell activation by secreting immunosuppressive cytokines such as IL-10 and TGF-β, induce a tolerogenic phenotype in dendritic cells, and promote the formation of a regulatory immune microenvironment (124, 125). Furthermore, experimental studies in KT models suggest that Tregs are closely linked to the suppression of pro-inflammatory Th17 responses and the control of cytotoxic CD8^+^ T cell-mediated damage (**Table 2**) (126). By modulating these effector cell populations, the Treg network can alleviate tubulointerstitial inflammation in kidney grafts and delay the process of chronic fibrosis remodeling (**Figure 2**) (127). Additionally, efforts to explore donor antigen-specific Tregs and engineered Tregs (such as CAR-Tregs) are advancing in preclinical settings, aiming to improve the precision of immune regulation and graft-homing capabilities (128). These experimental studies provide a solid theoretical foundation for using Tregs in KT tolerance induction. Based on these experimental findings, KT became one of the first solid organ transplants to initiate clinical translational research on Treg cell therapy.

From a translational medicine perspective, the focus of current research on Treg therapy in KT has gradually shifted from proof-of-concept safety toward optimizing cell product quality, enhancing antigen specificity, and developing combined immunoregulatory strategies that promote Treg survival and functional maintenance. On one hand, researchers continue to improve the *in vitro* preparation system of Tregs to enhance their phenotypic stability, suppressive activity, and batch-to-batch consistency (129). In parallel, the interaction between baseline immunosuppressive regimens and Treg biological behavior has increasingly gained attention. The differential effects of various immunosuppressive drugs on Treg survival and function (130) suggest that synergistic optimization of “cell therapy + drug modulation” will be a key direction for future clinical trial designs. Additionally, recent studies on Treg therapy in human KT have reported the emergence of unique immunoregulatory infiltrating cell characteristics in grafts from recipients treated with Tregs. These infiltrating cells are enriched with immune tolerance-related signals, including regulatory markers such as IKZF2, IL-10, PD-L1, and TIGIT (**Table 2**) (131). Unlike classical pro-inflammatory infiltrates, these findings provide new human evidence supporting the biological activity and translational relevance of Treg therapy in KT.

Against this translational background, the clinical development of Treg therapy in kidney transplantation has gradually progressed from early safety testing toward more precise and engineered approaches. The Treg Cell Transplantation (TRACT) trial, a landmark phase I study in the field of KT, systematically evaluated the use of autologous polyclonal expanded Tregs in living kidney transplant recipients for the first time, confirming the safety, feasibility, and *in vivo* persistence of this strategy, thus laying the groundwork for further research (52). Building on this, the ONE Study alliance, through a series of coordinated phase I and IIa clinical trials, further validated the safety of Treg infusion in a multi-center framework and demonstrated its potential clinical value in maintaining stable graft function and supporting immunosuppressive reduction (54). At the same time, researchers began exploring the application of donor antigen-specific Tregs in KT. Preliminary observations suggest that, compared to polyclonal Tregs, donor-specific formulations may achieve more precise immune regulation with lower cell doses (14), although related evidence is still in the early stages and requires validation through larger-scale, prospective studies. In this context, the ongoing STEADFAST trial represents an important step in the clinical translation of engineered Tregs in kidney transplantation. This first-in-human, phase I/IIa study is evaluating TX200-TR101, an autologous HLA-A2-specific CAR-Treg product, in HLA-A2-mismatched living donor kidney transplant recipients, with the goal of assessing its safety, tolerability, and potential to support graft-directed immune regulation (**Table 1**) (132).

In terms of safety, all published clinical studies of Treg therapy in KT to date have demonstrated good tolerability. Infusion-related adverse events have been generally rare and mostly mild, and no increased risk of serious infections or malignancies directly attributable to Treg treatment has been observed (**Table 1**) (17, 47, 133). Following infusion, a transient increase in the proportion of Tregs in peripheral blood is typically observed, accompanied by changes in immunoregulatory molecular profiles, indicating clear biological activity *in vivo* (**Table 1**) (131). However, similar to LT, a key bottleneck in Treg therapy for KT remains the lack of validated biomarkers that can reliably predict clinical tolerance and long-term graft outcomes. Closely integrating systematic immune monitoring with clinical endpoints to establish reproducible and verifiable predictive models will be critical for further advancing the application of Treg therapy in KT.

In summary, KT, as the most common type of SOT worldwide, faces the dual challenges of chronic allograft nephropathy and long-term immunosuppressive toxicity, providing a highly realistic clinical application scenario for Treg immunotherapy. A large body of basic research has elucidated the key role of Tregs in regulating the immune microenvironment of KT. Phase I/II clinical trials have confirmed the safety and feasibility of this therapy, with early evidence showing potential for reducing the immunosuppressive burden. With the ongoing development of engineered Tregs, precise immune monitoring, and combined immunoregulatory strategies, Treg therapy is expected to become an important tool in promoting the establishment of functional tolerance in future KT.

### 4.3 Heart transplantation

Compared to kidney and liver transplantation, heart transplantation (HT) presents unique immunological challenges, primarily due to the high incidence of chronic allograft vasculopathy (CAV) and limited spontaneous tolerance (**Figure 2**) (134). Therefore, Treg therapy in the field of HT is still in its early stages. However, preclinical studies have confirmed the biological feasibility of Treg therapy, and early clinical explorations are gradually revealing its safety and immunoregulatory effects.

A large number of animal studies have mechanistically demonstrated that Tregs play a critical role in immune regulation in HT. In murine allogeneic HT models, *in vivo* expansion or adoptive transfer of Tregs significantly prolongs graft survival, suppresses ACR, and markedly ameliorates CAV, a core pathological process that determines long-term prognosis (84). Mechanistic studies further indicate that, in HT models, IL-35–mediated immunoregulation promotes the accumulation and functional stability of intragraft Tregs, and its increase is closely associated with reduced infiltration of CD8^+^ cytotoxic T cells and downregulation of pro-inflammatory cytokines such as IFN-γ and TNF-α (**Table 2**) (135), highlighting the pivotal role of Treg-mediated immunoregulatory pathways in rejection suppression. Tregs exert immunosuppressive effects through the coordinated action of soluble mediators and contact-dependent mechanisms. For example, as discussed above, Tregs suppress alloimmune responses through multiple established contact-dependent and cytokine-mediated mechanisms. In heart transplantation, these regulatory effects are thought to contribute particularly to the control of graft inflammation and the attenuation of chronic allograft vasculopathy within the cardiac allograft microenvironment(**Figure 2**) (136). In recent years, engineered Tregs, particularly CAR-Tregs (84, 137) have shown enhanced antigen specificity and graft-homing capacity in animal models of HT, enabling more effective control of local inflammation and attenuation of intimal hyperplasia, and providing new experimental evidence for precision immunoregulation in HT.

While these engineered approaches show profound efficacy *in vivo*, human clinical data in heart transplantation are currently limited to observational reports and early safety validations. Compared with liver and kidney transplantation, clinical studies of Tregs in HT are markedly lagging, and large-scale phase I/II Treg therapy trials specifically designed for HT are still lacking. However, in the first reported case of thymus-derived Treg (thyTreg) therapy in a pediatric heart transplant recipient, autologous Tregs were generated from thymic tissue routinely removed during pediatric cardiac surgery and administered within an ongoing phase I/II clinical trial. In this case, with a 2-year follow-up, no severe adverse events clearly attributable to thyTreg infusion were observed, and the proportion of Tregs in peripheral blood was maintained or increased, indicating sustained biological activity *in vivo*. These findings support the tolerability of Treg cell therapy in this setting and suggest overall safety and feasibility in heart transplant recipients (**Table 1**) (138). Although the sample size was limited to a single case and therefore insufficient to evaluate clinical benefits with respect to acute rejection or CAV, these results provide important feasibility evidence for subsequent Treg clinical trials specifically designed for HT.

From a translational medicine perspective, the focus of current research on Treg therapy in HT is gradually shifting from the question of “whether it is safe and feasible” to “how to enhance efficacy and specificity.” At the same time, increasing attention is being paid to the interaction between baseline immunosuppressive regimens and Treg biological behavior in the field of HT. The effects of different immunosuppressive agents on Treg stability, migratory capacity, and suppressive function are emerging as key variables in clinical protocol design (139). The synergistic optimization of “cell therapy plus refined immunomodulation” is widely regarded as the core pathway to achieving breakthroughs in Treg therapy for HT. However, because HT requires long-term follow-up and the progression of CAV is often insidious, the evaluation of safety and efficacy relies particularly heavily on high-quality immune monitoring systems.

In summary, HT, characterized by a high incidence of CAV, limited intrinsic tolerance, and prominent complications associated with long-term immunosuppression, represents a transplant field in urgent need of innovative immunoregulatory strategies. Although its clinical translational progress currently lags behind that of liver and kidney transplantation, robust evidence from animal studies has clearly supported the critical role of Tregs in regulating acute rejection and chronic vasculopathy. Early human studies have also demonstrated preliminary safety and *in vivo* immunoregulatory potential. With advances in engineered Treg technologies, maturation of translational frameworks, and the establishment of predictive biomarkers, the clinical value of Tregs in controlling cardiac allograft rejection, delaying CAV progression, and reducing the lifelong burden of immunosuppression is expected to be more clearly defined in the future.

### 4.4 Lung transplantation

Lung transplantation (LuT) remains one of the most challenging forms of SOT in terms of long-term outcomes, as despite the use of modern immunosuppressive therapies, its median survival is still significantly lower than that of kidney or liver transplantation (140). Chronic lung allograft dysfunction (CLAD), risk of infection, and host-versus-graft injury constitute major obstacles (141–144). Regulatory T cell therapy has attracted considerable attention as a therapeutic strategy because of its ability to modulate host-versus-graft responses and promote tolerance within the unique immune environment of the lung. The pulmonary immune environment is inherently complex due to continuous exposure to environmental antigens entering via the airways, requiring robust local immune regulation and surveillance mechanisms (**Figure 2**). After transplantation, this environment may contribute to enhanced host-versus-graft responses and recruitment of effector lymphocytes (145, 146). Owing to their capacity to suppress effector T-cell responses while also modulating broader alloimmune and inflammatory networks, Treg cells could theoretically help counteract these processes if they can be efficiently delivered to and stably engrafted within lung tissue.

Compared with other organs, the lung is continuously exposed to environmental antigens, rendering the graft microenvironment more prone to immune activation and amplification of inflammation and therefore more highly dependent on regulatory immune networks (147). In immunological studies of LuT, Tregs are considered key factors in suppressing graft immune rejection and promoting immune tolerance. Recent studies have shown the presence of Foxp3^+^ regulatory T cells within lung allografts of transplant recipients, and these local Tregs may exert tissue-specific regulatory effects during the establishment of tolerance (148). Existing LuT studies have indicated that Treg dysfunction is associated with the progression of CLAD (149); however, systematic experimental evidence for adoptive Treg therapy in LuT models remains very limited and requires further investigation for validation. Mechanistic studies of Tregs in the context of LuT indicate that Tregs mediate immunoregulation through the production of anti-inflammatory cytokines such as IL-10, TGF-β, and IL-35, which contribute to the suppression of effector T cell responses (150). In inflamed lung allografts, enhanced Treg-associated signaling has been correlated with reduced Th1/Th17 effector-cell activity and decreased expression of pro-inflammatory cytokines (151), consistent with the concept that an optimal Treg/Teff balance supports immune regulation in lung tissue (**Figure 2**). In recent years, some investigators have explored strategies such as targeted organ delivery of Tregs during ex vivo lung perfusion to enhance Treg survival and functional activation within lung allografts (40). Furthermore, advanced genome-editing technologies have been applied to donor lungs in experimental settings. For example, CRISPR-mediated activation of IL-10 IL-10 in donor lungs has been proposed to upregulate this anti-inflammatory signaling, with the effect maintained after transplantation, highlighting the feasibility of enhancing graft immunoregulatory capacity through engineering approaches (152). These foundational studies provide solid experimental support for the application of Tregs as “local tolerance–inducing tools” in LuT. Moving from these experimental proofs of concept to clinical reality, translational research on Treg therapy in lung transplantation is markedly lagging behind other solid organs. To date, large-scale phase I/II clinical trials of Treg cell therapy specifically designed for lung transplant recipients are still lacking. Current human evidence mainly derives from two sources: first, the accumulated safety and immunological experience from Treg trials in other solid organ transplants; and second, exploratory translational studies focused on immune regulation in LuT. At present, only one clinical trial using Treg cell therapy to treat post-transplant rejection is registered on ClinicalTrials.gov (NCT00340951), and the results of this study have not yet been published (**Table 1**) (150). Although publicly available clinical efficacy data remain extremely limited, existing studies indicate that Tregs have clear biological plausibility in the immunological context of LuT, and that research at the design level is gradually progressing from “proof of concept” toward “clinical feasibility assessment.”

From a translational medicine perspective, the LuT field has also placed strong emphasis on optimizing the route and timing of administration. Compared with simple intravenous infusion, bronchoscopic or airway-local delivery of Tregs is considered more conducive to their engraftment within the lung and to the exertion of local immunoregulatory effects. At the same time, there is a broad consensus in the LuT community that Treg therapy is more likely to serve as an adjunctive immunomodulatory strategy to existing standard immunosuppressive regimens, rather than replacing these drugs in clinical practice in the short term (153, 154). With regard to safety, because the lung is one of the transplanted organs most susceptible to infection, Treg-associated immunoregulation must achieve a more refined balance between “tolerance induction” and “pathogen defense.” Another core challenge facing Treg therapy in LuT is the lack of reliable, validated biomarkers. Some studies have analyzed paired bronchoalveolar lavage samples and peripheral blood mononuclear cells by flow cytometry, demonstrating that combined sampling can more accurately reflect the post-transplant immunological landscape (**Table 2**) (149). When integrated with traditional clinical tools such as imaging and pulmonary function testing, these immunological parameters may enable the construction of a more comprehensive monitoring framework for patient stratification and outcome prediction. Moreover, with the increasing prominence of immune checkpoints, studies have reported that aberrant expression of immune checkpoint pathways may be involved in the mechanisms underlying CLAD progression (**Table 2**) (155). These preliminary data support the potential value of incorporating immune checkpoints together with Tregs into long-term immune monitoring frameworks, particularly for early identification and risk stratification of CLAD.

Overall, LuT remains one of the most challenging solid organ transplants in terms of long-term prognosis, with CLAD, infection, and chronic inflammatory responses serving as the core bottlenecks limiting improvements in survival. A substantial body of preclinical research has confirmed the critical role of Tregs in regulating the immune homeostasis of lung allografts at both the mechanistic and functional levels, but its clinical translation is still in the early stages. In the future, with the development of engineered Treg technologies, local delivery strategies, and multi-omics immune monitoring systems, LuT is expected to gradually establish Treg therapy regimens tailored to its unique immune environment. Continuing to advance safety assessments and mechanism-driven clinical trials will be key to clarifying the true value of Tregs in improving long-term outcomes in LuT.

## 5 Challenges and future perspectives of Treg therapy

Although early clinical trials have reported compelling biological rationale and encouraging safety profiles, Treg-based therapies have not yet achieved routine clinical application in SOT. This translational gap reflects multiple unresolved issues at the biological, technical, and clinical levels that collectively limit the robustness and scalability of these approaches. From a translational standpoint, these limitations can be understood as recurrent points of failure arising at different stages along the therapeutic continuum, spanning product attributes, *in vivo* delivery, phenotypic stability, target engagement, and endpoint selection. Recognizing these recurring vulnerabilities can improve the interpretability of both positive and negative trial signals and help distinguish true biological limitations from avoidable design or measurement constraints. At the cellular level, the long-term phenotypic stability, lineage fidelity, and functional durability of ex vivo–expanded Tregs remain incompletely controlled (51, 156, 157). After transplantation, the inflammatory microenvironment—characterized by IRI (158), pro-inflammatory cytokines (22), and strong alloantigenic stimulation (159)—may impair FOXP3 expression, metabolic adaptability, and suppressive capacity (160, 161), thereby leading to variability in efficacy and potential safety concerns. Importantly, current uncertainty regarding therapeutic efficacy is often not simply attributable to “insufficient dose”, but more likely arises from the inability of effective Tregs to achieve adequate, stable, and targeted residency and function within critical spatiotemporal windows. Accordingly, “delivery deficit” (failure to reach graft/draining lymphoid compartments) and “identity drift” (loss of suppressive program under inflammatory stress) represent two mechanistically plausible failure modes that can coexist and jointly reduce apparent efficacy even when products are numerically adequate in peripheral blood. In this context, the practical limitations of most clinical protocols that still rely on polyclonal Treg products are further magnified, including insufficient donor-antigen specificity, limited graft-homing efficiency, and marked heterogeneity in *in vivo* persistence. This can be viewed as a “targeting mismatch,” in which broad immunoregulation is expected to control highly focused donor-reactive pathways, often under conditions of high inflammatory load. These constraints necessitate the use of higher cell doses to compensate for the deficit in “sustained presence of effective cells at the correct site,” thereby increasing the risk of systemic immunomodulation rather than achieving localized tolerance (162, 163). Such considerations underscore that the effective therapeutic dose should be defined by functional and spatial exposure at sites of alloimmune regulation, not solely by the infused cell number.

In addition to biological limitations, translational progress is also hindered by significant manufacturing and regulatory challenges. Current GMP-compliant expansion protocols are costly, time-consuming, and exhibit substantial donor-to-donor variability, while standardized definitions of product potency and release criteria relevant to clinical outcomes are still lacking (164, 165). This makes cross-center efficacy comparisons and large-scale implementation more difficult. In other words, whether Treg therapy can truly achieve “replicability” largely depends on the establishment of key quality attributes and potency readouts that are regulatory-approved and linked to mechanisms and outcomes. In this setting, limited transparency and harmonization in CMC—particularly batch-to-batch variability in purity, stability, and functional potency—can obscure dose–response relationships and undermine cross-trial comparability. Without these, positive signals from early trials are unlikely to translate into widely applicable therapeutic paradigms. Furthermore, the interaction between graft-derived Tregs and conventional immunosuppressive regimens adds another layer of complexity. Calcineurin inhibitors directly disrupt the IL-2-dependent signaling pathway necessary for Treg survival and stability (89, 166), while lymphocyte-depleting induction therapies during the transplant period, together with corticosteroid exposure and innate inflammatory signals, remodel the host immune environment and may delay the re-establishment of regulatory immune networks, potentially impairing graft chimerism, Treg survival, and regulatory network formation (167–170). This “pharmacologic antagonism” highlights that Treg therapy should not be evaluated as an add-on in a fixed background regimen, but rather within an immunomodulatory context deliberately aligned with Treg biology. Therefore, the key for the future lies not in simply adding cell products to existing regimens but in redesigning the perioperative immune modulation context around the biological needs of Tregs. This would include controlling inflammation peaks, supporting the IL-2 axis, and optimizing immunosuppressive drug windows to improve the biological success rate of cell therapies and reduce variability. One of the major reasons exacerbating these challenges is the lack of validated biomarkers and clinically meaningful alternative endpoints (171), which could be used to define, predict, and longitudinally track tolerance. This limitation can be conceptualized as “measurement failure,” where the absence of prespecified target-engagement and proof-of-biology readouts makes early-phase trials difficult to interpret and slows rational iteration. This limitation restricts proper patient stratification, adaptive trial designs, and regulatory assessments. Without actionable alternative endpoints, Treg therapy will struggle to transition from “safe and feasible” to “efficacious and demonstrable,” and even harder to establish decision frameworks for immunosuppressive tapering. Therefore, biomarker-driven evaluation systems should be considered as an essential component of the translational infrastructure as the cell products themselves. At minimum, a compact metric set should capture post-infusion exposure (persistence and, where feasible, evidence of target-site presence), *in vivo* stability as a longitudinal trajectory, and target engagement reflected by modulation of donor-reactive immune responses; integration of humoral alloimmunity (e.g., donor-specific antibodies) alongside cellular readouts is also important to capture antibody-mediated risk. Mechanistic trajectories should be linked to organ-appropriate injury/remodeling surrogates to bridge biological activity with clinically meaningful consequence, particularly in early-phase studies where hard endpoints are insensitive.

Building on the above considerations, these bottlenecks have collectively catalyzed a paradigm shift, driving the development of next-generation precision Treg therapies aimed at overcoming the limitations of first-generation approaches. Advances in cell engineering technologies are promoting the development of donor antigen–specific Tregs, including TCR-transgenic and CAR–engineered Tregs, which enhance specificity, activity, and graft-targeting efficiency while minimizing off-target immunosuppression (172, 173). In failure-mode terms, these approaches are positioned to mitigate targeting mismatch and reduce the need for supraphysiologic dosing by increasing donor-focused potency at relevant sites. In parallel, epigenetic, metabolic, and genetic optimization strategies are being explored to reinforce lineage stability, increase resistance to inflammatory destabilization, and improve *in vivo* persistence (77, 174–176). These strategies directly address identity drift and potency–inflammation mismatch by strengthening fitness under post-transplant stress. Future clinical success will depend on integrating Treg therapy into rationally designed immunomodulatory regimens. Centered on antigen targeting, reinforced stability, and closed-loop monitoring, such strategies would actively support regulatory networks through customized immunosuppression, adjunctive cytokine therapies, and tolerance-oriented preconditioning. Importantly, “closed-loop monitoring” requires that biomarker hypotheses and sampling schedules be prespecified to demonstrate target engagement and to guide risk-controlled immunosuppression minimization rather than empirical tapering. This approach aims to achieve stepwise immunosuppressive minimization under controlled risk, rather than pursuing abrupt and complete drug withdrawal. Equally important is the integration of high-dimensional immune monitoring platforms, including single-cell multi-omics analyses, TCR repertoire tracking, spatial tissue profiling, and donor-derived cell-free DNA detection, to establish biomarker-driven frameworks for patient selection, dose optimization, and early identification of tolerance establishment or immune escape (**Table 2**). Such platforms are particularly valuable when anchored to a minimal, harmonized core dataset (product descriptors, background regimen, key biomarkers, and endpoint definitions), enabling comparability across centers and accelerating cumulative learning. As scalable manufacturing technologies, automated production systems, and harmonized regulatory standards mature, these advances are expected to transform Treg therapy from proof-of-concept interventions into reproducible precision immunotherapies. Ultimately, the successful convergence of cell engineering, systems immunology, and clinical trial innovation may drive SOT away from chronic, non-specific pharmacologic immunosuppression toward more durable and targeted immune tolerance.

## 6 Conclusion

Treg therapy represents a paradigm shift in the field of SOT, with the potential to overcome the limitations of chronic, non-specific immunosuppression and to achieve durable antigen-specific immune tolerance. Although early clinical trials have demonstrated feasibility and short-term safety, limitations related to lineage stability, antigen specificity, *in vivo* persistence, manufacturing scalability, and biomarker-guided implementation continue to constrain clinical translation. Rapid advances in cell engineering, immune monitoring, and precision immunomodulation are now driving the development of next-generation Treg products with enhanced antigen specificity and optimized function. If these innovations can be effectively integrated with rational clinical trial design, Treg therapy is poised to evolve from an experimental intervention into a reproducible, mechanism-based strategy for long-term transplant tolerance.
